# Schuylkill Valley Veterinary Medical Association

**Published:** 1896-07

**Authors:** U. G. Fridirici

**Affiliations:** Secretary


Secretary.

### SCHUYLKILL VALLEY VETERINARY MEDICAL ASSOCIATION.

THE Association held its annual meeting in the parlors of Hotel Woll, Pottsville, Pa., June 17, 1896. The meeting was called to order by the President, Dr. S. G. Burkholder, at 10 A.M. On roll-call the following members responded: Burkholder (President), Sallade, Faughman, Fridirici, Noack, Snyder, Kershner, Bieber, and Moyer. The minutes of the previous meeting were read and approved.

Dr. W. H. Yingst, of Harrisburg, Pa., was duly elected as a member of the Association.

Th following officers were elected by ballot for the ensuing year: President, Dr. S. G. Burkholder, Denver, Pa.; Vice-President, Dr. W. H. Moyer, Elizabethville, Pa.; Corresponding Secretary, Dr. Otto Noack, Reading, Pa.; Recording Secretary, Dr. U. G. Fridirici, Tamaqua, Pa.; Treasurer, Dr. Frank McCarthy, Pottsville, Pa.; Trustees, Drs. J. W. Sallade, Pottsville, Pa.; P. J. Kershner, Fleetwood, Pa.; U. S. Bieber, Kutztown, Pa.

Dr. Otto Noack was appointed delegate to the State Veterinary Association, which meets in Reading, Pa., in September.

Dr. J. W. Sallade was appointed delegate to the National Veterinary Association, which meets in Buffalo, N. Y.

The following papers were read and discussed: "Mange," by Dr. U. S. Bieber. "Castration," by Dr. W. H. Moyer. "Emphysema," by Dr. Otto Noack.

*Report of Case.* Dr. U. S. Bieber was called to see a case May 8th; found it to be a bay gelding, twelve years old, suffering from enlargement of right side of cheek. He at once diagnosed a case of salivary calculi, and advised an operation, which was performed; upon the removal of calculus found it to weigh eight ounces. A complete recovery was the result.

Papers for next meeting: Dr. J. W. Sallade, "The Social Position of the Veterinarian." Dr. J. C. Faughman, "Tetanus." Dr. A. Pottenger, "Pneumonia." Dr. W. H. Yingst, "Shoeing." Dr. U. G. Fridirici, "Periodic Ophthalmia."

Meeting adjourned at 3 P.M. Next meeting to be held in September in the city of Reading.

DR. U. G. FRIDIRICI,
Secretary.

